# *Neurospora* COP9 Signalosome Integrity Plays Major Roles for Hyphal Growth, Conidial Development, and Circadian Function

**DOI:** 10.1371/journal.pgen.1002712

**Published:** 2012-05-10

**Authors:** Zhipeng Zhou, Ying Wang, Gaihong Cai, Qun He

**Affiliations:** 1 State Key Laboratory of Agrobiotechnology, College of Biological Sciences, China Agricultural University, Beijing, China; 2 National Institute of Biological Sciences, Beijing, China; Yale University, United States of America

## Abstract

The COP9 signalosome (CSN) is a highly conserved multifunctional complex that has two major biochemical roles: cleaving NEDD8 from cullin proteins and maintaining the stability of CRL components. We used mutation analysis to confirm that the JAMM domain of the CSN-5 subunit is responsible for NEDD8 cleavage from cullin proteins in *Neurospora crassa*. Point mutations of key residues in the metal-binding motif (EX_n_ HXHX_10_ D) of the CSN-5 JAMM domain disrupted CSN deneddylation activity without interfering with assembly of the CSN complex or interactions between CSN and cullin proteins. Surprisingly, CSN-5 with a mutated JAMM domain partially rescued the phenotypic defects observed in a *csn-5* mutant. We found that, even without its deneddylation activity, the CSN can partially maintain the stability of the SCF^FWD-1^ complex and partially restore the degradation of the circadian clock protein FREQUENCY (FRQ) *in vivo*. Furthermore, we showed that CSN containing mutant CSN-5 efficiently prevents degradation of the substrate receptors of CRLs. Finally, we found that deletion of the CAND1 ortholog in *N. crassa* had little effect on the conidiation circadian rhythm. Our results suggest that CSN integrity plays major roles in hyphal growth, conidial development, and circadian function in *N. crassa*.

## Author Summary

Cullin-RING E3 ubiquitin ligases (CRLs) play important roles in regulating a wide range of processes, such as signal transduction, transcription, cell cycle progression, circadian rhythm, and development, via the ubiquitin-proteasome pathway. The activity and stability of CRLs is precisely controlled by the COP9 signalosome (CSN), an evolutionarily conserved multisubunit protein complex. Under the control of the CSN, CRL activity can be either downregulated via cleavage of NEDD8 (an ubiquitin-like protein) from cullin proteins (deneddylation) or preserved by maintaining the stability of CRL components. We generated point mutations of key residues in the JAMM domain of the CSN-5 subunit to disrupt CSN deneddylation activity, thereby creating a series of mutants containing the intact CSN complex but lacking deneddylation activity. Surprisingly, hyphal growth, conidial development, circadian rhythm, and stability of the SCF^FWD-1^ complex in these CSN-5 point mutants were comparable to that observed in wild-type *N. crassa*. Furthermore, we showed that CSN containing mutant CSN-5 efficiently prevents degradation of the substrate receptors of CRLs. Finally, deletion of the *N. crassa* ortholog of CAND1 (cullin-associated NEDD8-dissociated protein 1) had little effect on conidial development and the circadian clock. Our results suggest that the integrity of the CSN is important for growth and development in *N. crassa*.

## Introduction

The COP9 signalosome (CSN) is an evolutionarily conserved multifunctional complex in eukaryotes; it is composed of eight subunits (CSN1–CSN8) in plants and mammals [1]. The CSN was initially discovered to be an important regulator of photomorphogenesis in *Arabidopsis thaliana* [2] and was later found to participate in a wide range of processes [1], [3]. The CSN potentially influences these cellular pathways by regulating the activity of cullin-RING ubiquitin ligases (CRLs, e.g., CRL1, CRL3, and CRL4 complexes in most eukaryotes) [3], [4], [5]. CRLs are a big family of ubiquitin ligases that share a common cullin/RING-E2 module [6], [7], [8]. They are necessary for substrate ubiquitination in a cascade of enzymatic reactions involving E1, E2, and E3 [9]. Under the control of the CSN-regulated ubiquitin-proteasome pathway, cells coordinate the expression of an array of genes involved in the regulation of growth and development in order to respond to environmental signals, such as light, temperature, and changes in nutrient conditions [1], [10]. Loss-of-function mutations in CSN subunits result in dysfunction of hundreds of CRLs [3], which explains the pleiotropic phenotypes observed in CSN mutants [1], [3], [7].

In 2002, Deshaies and his colleagues first described that the CSN-5 metalloprotease (JAMM) motif is required for removing the ubiquitin-like protein NEDD8 from Cul1 [11]. Later studies confirmed that the isopeptidase activity of the CSN complex is responsible for cullin deneddylation in eukaryotes [1], [3], [4], [5], [12]. In this process, the CSN binds to CRL E3 ligase and cleaves NEDD8 from cullins via the catalytic activity of its CSN-5 subunit, and then inhibits CRL activity [5], [13], [14], [15]. Thus, the deneddylation activity of CSN requires the metalloprotease motif located in the CSN-5 subunit and the functional core subunits of the CSN [11], [16]. However, CSN-5–dependent metalloprotease activity is not essential in *Schizosaccharomyces pombe*, as no obvious phenotype was detected in *csn-5* deletion strains [17], [18].

The physiological importance of CSN deneddylation activity in development and cell differentiation was examined in *Drosophila melanogaster*, in which the lethality of *csn-5^Δ/Δ^* animals was rescued by expression of a CSN-5 transgene but no adult flies were recovered upon equivalent expression of CSN-5 (D148N) (loss of deneddylation activity) [11], [19]. In CSN-5-downregulated HeLa cells, however, the accelerated degradation of c-Jun was rescued equally by over-expression of either the JAMM domain mutant CSN-5D151N or wild-type CSN-5 [20]. These results suggest that the requirement for neddylation/deneddylation cycle of cullins is not absolutely necessary during normal growth and certain developmental stages. In plants, genetic studies suggest that although neddylation/deneddylation cycle is not absolutely necessary during early embryonic development and germination, it is required during seedling establishment and the later developmental stages [12], [21]. In *Aspergillus nidulans*, deletion of *csnE/csn-5* or mutation in JAMM domain results in a block in fruiting body formation at the primordial stage, with a few other observed phenotypic changes, such as light-dependent signaling [22], [23]. Although deneddylation is a major activity of the CSN, it alone cannot explain all of the phenomena described above. These observations raise the possibility that the CSN may have other functional activities in addition to its deneddylation activity.

Recent genetic evidence suggest that the CSN has one additional major function: it controls the stability of CRL ubiquitin ligases *in vivo* by mediating assembly/disassembly of CRL complexes and by protecting substrate receptors in CRLs from degradation [3], [24], [25], [26], [27], [28]. A recent structural and biochemical study showed that the protective effect of the CSN on DDB2 and CSA autoubiquitination in CRL4 complexes does not require CSN-5–mediated deneddylation activity [29]. However, both of the CSN activities occur when CSN associates with cullins in CRL E3 complexes. Furthermore, there is also a tight correlation between CSN deneddylation activity and the ability of the complex to modulate the stability of CRLs [3]. Thus, it is difficult to determine which function is more important for growth and development through regulation of CRL activity, or how these two functions cooperate with each other in regulating CRL dynamicity in eukaryotes. In *A. thaliana*, the MPN (Mpr-Pad1-N-terminal domain) subunits CSN-5 and CSN-6 are essential for the structural integrity of the CSN holocomplex [12]. Several studies have shown that point mutations in the JAMM metal-binding site of CSN-5 do not interfere with the proper assembly of CSN complexes in *S. pombe*, *A. thaliana*, and *A. nidulans* [11], [21], [23]. In *N. crassa*, CSN-5 is not an essential gene; the deletion mutant can survive, and displays obvious growth and developmental defects, making it an excellent model system for investigating the distinctions between the deneddylation and CRL complex assembly/disassembly functions of the CSN [16].

The CSN takes part in a wide range of cellular and developmental processes in *N. crassa*, including hyphal growth, conidial formation, light and temperature responses, and circadian clock function [16], [24]. To further investigate its biological function *in vivo*, we created a series of point mutations in the JAMM metal-binding motif of the CSN-5 subunit to disrupt the deneddylation activity of the CSN complex. In those mutant strains, the integrity of the CSN and its interactions with Cul1 and Cul4 were not affected. Surprisingly, mutated CSN-5 almost retained the ability to restore the phenotypic defects of a *csn-5^KO^* strain and partially maintained the stability of the SCF^FWD-1^ complex, which was able to carry out degradation of the clock protein FREQUENCY (FRQ) *in vivo*. Moreover, the stability of four other substrate receptors of CRLs can be efficiently restored by the CSN containing mutant CSN-5. However, deletion of the CAND1ortholog in *N. crassa* had little effect on conidiation circadian rhythm and the degradation of FRQ. Our results suggest that the integrity of CSN plays major roles in hyphal growth, conidial development, and circadian function in *N. crassa*.

## Results

### Mutations within the CSN-5 JAMM metal-binding site abolish CSN–mediated deneddylation of cullins

**Figure 1:**
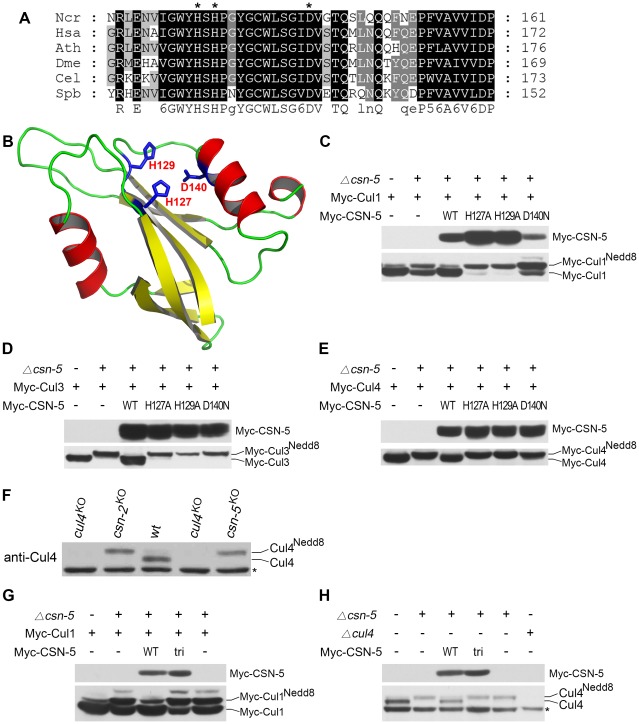
Mutations within the JAMM motif of CSN-5 abolish CSN–mediated deneddylation activity for Cul1, Cul3, and Cul4. (A) Amino acid alignment of conserved JAMM motifs of CSN-5 homologs from *Neurospora crassa* (Ncr), *Homo sapiens* (Hsa), *Arabidopsis thaliana* (Ath), *Drosophila melanogaster* (Dme), *Caenorhabditis elegans* (Cel), and *Schizosaccharomyces pombe* (Spb). (B) Predicted structure of the *N. crassa* CSN-5 JAMM domain. The structure was generated by the SWISS-MODEL using the structure of the pre-mRNA splicing factor Prp8 as template (PDB accession number 2P8R), and the functional sites (His127, His129, and Asp140) were mapped according to the structure alignment with the AfJAMM structure (PDB accession number 1R5X). (C–E) Western blot analyses with c-Myc antibody of expression profiles of Myc-Cul1 (C), Myc-Cul3 (D), and Myc-Cul4 (E) in the wild-type strain, *csn-5^KO^*, and CSN-5 complementation strains. (F) Western blot analysis showing the expression of Cul4 in the wild-type, *cul4^KO^*, and *csn-5^KO^* strains. (G) Western blot analyses with c-Myc antibody of expression profiles of Myc-Cul1 in the wild-type strain, *csn-5^KO^*, and *csn-5^KO^*, pcsn-5-Myc-CSN-5 or *csn-5^KO^*, pcsn-5-Myc-CSN-5tri complementation strains. (H) Western blot analyses showing the expression of endogenous Cul4 in the wild-type strain, *csn-5^KO^*, *csn-5^KO^*, pcsn-5-Myc-CSN-5 or *csn-5^KO^*, pcsn-5-Myc-CSN-5tri complementation strains. The asterisk indicates a nonspecific cross-reacted protein band recognized by our Cul4 antiserum (in F and H).

The *N. crassa* genome encodes seven COP9 signalosome subunits (CSN-1–CSN-7) [16], [24]. Several studies have shown that the JAMM metal-binding sites in the MPN domain of CSN-5 are required for metalloprotease activity in the CSN [11], [21], [23]. When the CSN-5 protein sequence was used in a BLAST search against protein databases, a highly conserved MPN domain in the *N. crassa* CSN-5 subunit was identified. As shown in Figure 1A, three conserved residues corresponding to His127, His129, and Asp140 lie within the putative metal-binding motif (EX_n_ HXHX_10_ D) of the *N. crassa* CSN-5 JAMM domain. To determine whether these conserved residues form the metalloprotease-like active site of JAMM, we used the JAMM domain of *Archaeoglobus fulgidus* as a template to generate the tertiary structure of *N. crassa* CSN-5 [30]. Because of the low similarity between these two JAMM domains, the generated structure was poor. Thus, we instructed SWISS-MODEL to automatically select a template protein for generating the structure of *N. crassa* CSN-5 [31]. SWISS-MODEL selected the pre-mRNA splicing factor Prp8 as template (Protein Data Bank [PDB] accession number 2P8R) for *N. crassa* CSN-5. The functional sites were mapped into predicted structure according to the structural alignment with AfJAMM (PDB accession number 1R5X). As shown in Figure 1B, His127, His129, and Asp140 within EX_n_ HXHX_10_ D of the *N. crassa* CSN-5 JAMM corresponded to the putative metal-binding motif as metalloprotease-like active site in AfJAMM [30], [32].

To confirm the contribution of CSN-5 to CSN-mediated deneddylation of cullins, we mutated these three highly conserved amino acids (H127A, H129A or D140N) using site-directed mutagenesis. We then introduced quinic acid (QA)–inducible Myc-tagged wild-type CSN-5 or one of the three mutant CSN-5 constructs into a *csn-5^KO^* strain expressing Myc-Cul1 protein. As shown in Figure 1C, Myc-CSN-5, Myc-CSN-5H127A, Myc-CSN-5H129A, and Myc-CSN-5D140N were expressed in the *csn-5^KO^* strains in the presence of QA. Expression of Myc-tagged wild-type CSN-5 in the *csn-5^KO^* strain resulted in a decrease in hyperneddylated Cul1 to the level of the wild-type strain (Figure 1C), indicating that the Myc-tagged CSN-5 protein was functional for CSN deneddylation activity. In contrast, expression of mutant CSN-5 (H127A, H129A, or D140N) failed to decrease the hyperneddylated Cul1 in the *csn-5^KO^* strain (Figure 1C). Similarly, hyperneddylation of Cul3 (Figure 1D) and Cul4 (Figure 1E) in the *csn-5^KO^* strain was rescued by expressing the Myc-tagged wild-type CSN-5, but not by any of the mutated Myc-CSN-5s. This indicates that the metal-binding motif of JAMM is essential for CSN-mediated deneddylation of cullins.

Because all of the Cul3 and Cul4 was neddylated while not all of the Cul1 was neddylated in the *csn-5^KO^* strain and *csn-5^KO^* strains complemented by JAMM-domain mutant CSN-5, we rechecked Cul1 modification in the *csn* mutants. As shown in Figure S1, c-Myc antibody detected three specific protein bands in first generation of *csn-5^KO^* or *csn-6^KO^* transformants and two specific bands in the *csn-1^KO^* transformants. In most positive transformants, there was slightly less unneddylated Cul1 than neddylated Cul1, but the signal remained strong. This is different from the studies in yeast, plants, and fruit fly in which deletion of *csn-5* results in hyperneddylation of Cul1 [11], [21], [33]. Possible explanations are that *N. crassa* genome codes for another deneddylase in addition to CSN complex or there is large amount of newly synthesized Cul1 proteins. We next examined the neddylation of Cul4 using a polyclonal antibody that recognizes the N terminus of *N. crassa* Cul4. As shown in Figure 1F, only the neddylated Cul4 was detected in *csn-5^KO^* strain, while in the wild-type strain, most of the detected Cul4 was unneddylated. Next, we transferred endogenous *csn-5* promoter-driven constructs of either wild-type CSN-5 or CSN-5 with JAMM triple point mutations (H127A, H129A, and D140N) (hereafter referred to as CSN-5tri) into a *csn-5^KO^* strain expressing Myc-Cul1 protein. Myc-CSN-5 and Myc-CSN-5tri were expressed in the *csn-5^KO^* strains (Figure 1G). Similar to what we observed in *csn-5^KO^* expressing CSN-5 with a single point mutation (Figure 1C), expression of the CSN-5tri failed to decrease the hyperneddylation of Cul1 in the *csn-5^KO^* strain (Figure 1G) as well. Interestingly, the amount of unneddylated Cul1 in *csn-5^KO^* strains expressing either single (Figure 1C) or triple (Figure 1G) point mutant CSN-5 was less than that in a *csn-5^KO^* strain. Furthermore, expression of CSN-5tri in the *csn-5^KO^* strain also failed to decrease hyperneddylated Cul4 to the levels observed in the wild-type or *csn-5^KO^* strain complemented with wild-type CSN-5 (Figure 1H). Taken together, these data confirm that the JAMM domain metal-binding motif of *N. crassa* CSN-5 is essential for the deneddylation activity of the CSN.

### The rescue of growth and developmental defects in the *csn-5^KO^* strain by CSN-5 with JAMM domain mutations

**Figure 2:**
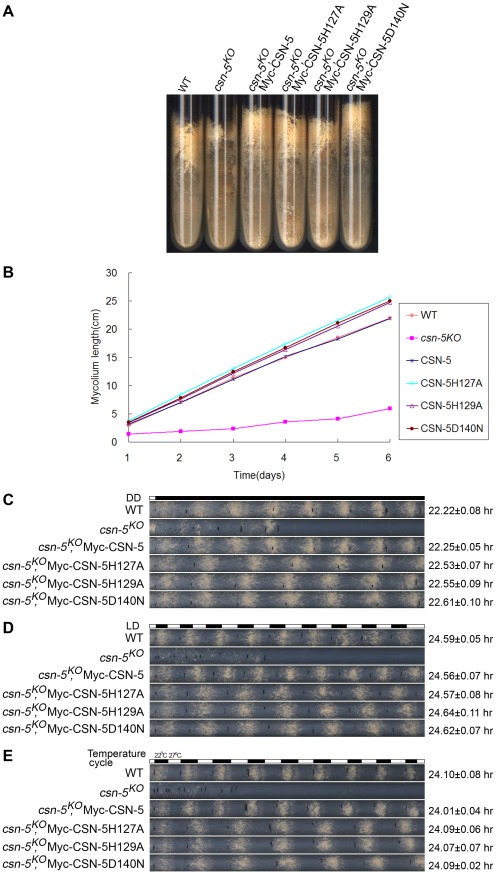
Rescue of growth and developmental defects in the *csn-5^KO^* strain by the expression of JAMM-motif mutant CSN-5. (A) Wild-type, *csn-5^KO^*, and the different CSN-5 complementation strains growing on slants containing QA. *csn-5^KO^* strains produced significantly less conidia and aerial hyphae than wild-type or CSN-5 complementation strains. (B) Growth rates of the wild-type, *csn-5^KO^*, and CSN-5 complementation strains, measured at 25°C by race tube assays in constant darkness after 1 d of light treatment. (C–E) Rescue of conidiation rhythms in the different CSN-5 complementation strains, measured by race tube assay in dark–dark (C), light–dark (D), and temperature cycles (E). At least four replicates were tested under each condition. Black lines indicate the growth fronts every 24 h.

To examine whether the JAMM metal-binding site of CSN-5 functions in growth and development, we analyzed the phenotypes of the *csn-5^KO^* strain expressing either Myc-tagged wild-type or mutant CSN-5. On minimal slants with QA, the *csn-5^KO^* strain produced fewer aerial hyphae and conidia than the wild-type strain (Figure 2A). Expression of wild-type CSN-5 in the *csn-5^KO^* strain restored aerial hyphal growth and conidial formation to levels similar to those in the wild-type strain (Figure 2A). Surprisingly, when *csn-5^KO^*, Myc-CSN-5H127A; *csn-5^KO^*, Myc-CSN-5H129A; and *csn-5^KO^*, Myc-CSN-5D140N strains (hereafter referred to as *csn-5^H127A^*, *csn-5^H129A^*, and *csn-5^D140N^*, respectively) were grown in minimal slants containing QA, the transformants exhibited hyphal formation and conidiation that were the same as the wild-type strain and the *csn-5^KO^*, Myc-CSN-5 strain (Figure 2A). We next measured the growth rates of the wild-type strain, the *csn-5^KO^* strain, and the transformants by race tube assay in constant darkness. Interestingly, the growth of *csn-5^H127A^*, *csn-5^H129A^*, and *csn-5^D140N^* strains was slightly faster than that of the wild-type strain (approximately 4.2 cm per day vs. 3.7 cm per day, respectively) and the *csn-5^KO^*, Myc-CSN-5 strain (Figure 2B). These results suggest that these CSN-5s with a point mutation within the JAMM metal-binding motif function similarly as the wild-type CSN-5 on *N. crassa* growth and conidiation. In QA-containing race tubes, the conidiation rhythms of the *csn-5^H127A^*, *csn-5^H129A^*, and *csn-5^D140N^* strains (a period of about 22.5 h) were pretty much (only slightly longer) to those of the wild-type and *csn-5^KO^*, Myc-CSN-5 strains (a period about 22.2 h) (Figure 2C) in constant darkness after light entrainment. To characterize the effect on light response of each CSN-5 point mutation, we further examined the light-entrained conidiation rhythm of each *csn-5^KO^* transformant during light–dark (LD) cycles (12 h light/12 h dark). As shown in Figure 2D, although the LD cycles entrained the conidiation rhythm of the *csn-5^KO^* strains expressing wild-type CSN-5 or mutant CSN-5, however, the conidiation bands of the *csn-5^H127A^*, *csn-5^H129A^*, and *csn-5^D140N^* strains were broader than those of the wild-type and *csn-5^KO^*, Myc-CSN-5 strains. Similarly, 12 h 27°C/12 h 22°C temperature cycles entrained the conidiation rhythm of the *csn-5^H127A^*, *csn-5^H129A^*, and *csn-5^D140N^* strains, but not the patterns of conidiation bands (Figure 2E). Taken together, these results suggest that point mutations within CSN-5 are functional in growth and conidiation, and partially functional in circadian rhythm, light response, and temperature-entrained clock process.

### JAMM domain mutations do not disrupt CSN complex integrity or its interactions with Cul1 and Cul4

**Figure 3:**
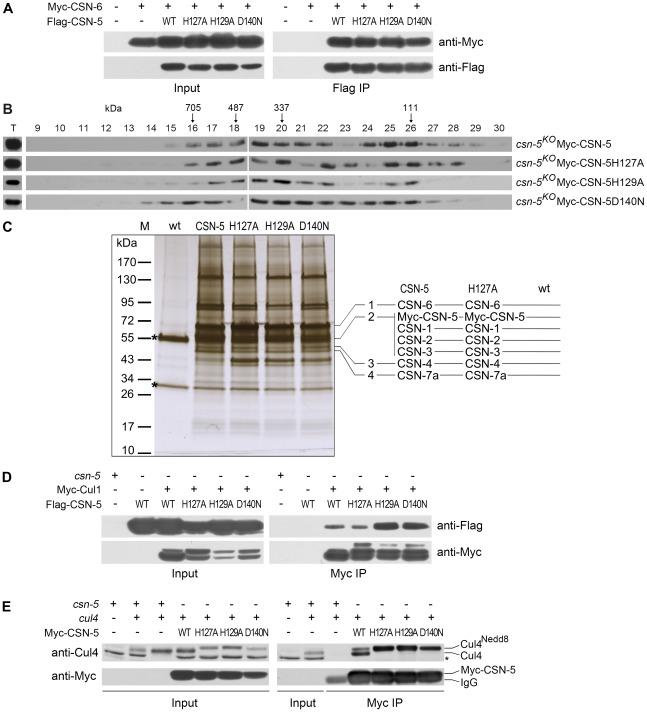
Point mutations do not disrupt integrity of the CSN or interactions of the CSN with Cul1 and Cul4. (A) Immunoprecipitation assays confirming interactions between different versions of Flag-CSN-5 and Myc-CSN-6. Wild-type strain and wild-type strain expressing Myc-CSN-6 were used as negative controls. (B) The Myc-CSN-5, Myc-CSN-5H127A, Myc-CSN-5H129A, or Myc-CSN-5D140N in *csn-5^KO^* properly incorporates into CSN complex. (C) Silver-stained SDS-PAGE showing the two-step purification of Myc-His-CSN-5, Myc-His-CSN-5H127A, Myc-His-CSN-5H129A, or Myc-His-CSN-5D140N in the *csn-5^KO^* strains. A wild-type strain was used as the negative control. CSN subunits identified by mass spectrometry analysis in products of Myc-His-CSN-5 or Myc- His-CSN-5H127A are indicated. Asterisks indicate the two IgG bands. (D) Immunoprecipitation assays confirming the interaction between different versions of Flag-CSN-5 and Myc-Cul1. (E) Immunoprecipitation assays showing the interaction between different versions of Myc-CSN-5 and endogenous Cul4. The asterisk indicates a nonspecific cross-reacted protein band recognized by our Cul4 antiserum.

The loss of deneddylation activity of the JAMM domain mutations may be due to the disruption of the CSN complex. To examine this, we tested the interactions between the CSN-6 subunit and wild-type or mutant CSN-5s. Myc-tagged CSN-6 was co-expressed with Flag-tagged CSN-5 or mutant CSN-5 proteins in *csn-5^KO^* strains. As shown in Figure 3A, the Flag-tagged CSN-5 strongly interacted with Myc-tagged CSN-6 in an immunoprecipitation reaction, suggesting that they were both in the intact CSN complexes. As expected, the Flag antibody pulled down the Myc-tagged CSN-6 protein in the *csn-5^KO^* strain co-expressing Myc-CSN-6 and each of the mutant Flag-CSN-5 proteins (Figure 3A), similar to what was observed in the *csn-5^KO^* strain co-expressing CSN-6 and wild-type CSN-5. This result indicates that the point mutations within the CSN-5 JAMM metal-binding motif did not affect the interactions between the CSN-5 and CSN-6 subunits and those two MPN proteins within PCI (Proteasome, COP9, eukaryotic Initiation factor 3) complexes may form dimers. To further examine whether Myc-His-tagged CSN-5 point mutants are incorporated into a larger molecular mass CSN complex, we performed gel filtration and followed by Western blot analysis. As shown in Figure 3B, like wild-type CSN-5, CSN-5H127A, CSN-5H129A, and CSN-5D140N fusion proteins were eluted in larger molecular mass fractions, suggesting that each of the Myc-tagged CSN-5 point mutants can be incorporated into the intact CSN complex.

Using protein affinity purification followed by Mass Spectrometry analysis, we further examined whether the CSN complex is properly assembled with CSN-5 point mutants. Myc-His-tagged CSN-5H127A, CSN-5H129A, CSN-5D140N, or wild-type CSN-5 was purified on a nickel column followed by immunoprecipitation with a c-Myc monoclonal antibody. As shown in Figure 3C, similar immunoprecipitated protein profiles were detected in the Myc-His-CSN-5H127A, Myc-His-CSN-5H129A, Myc-His-CSN-5D140N, and Myc-His-CSN-5 (wild-type CSN-5) samples, but not in the wild-type strain (negative control). Liquid chromatography–mass spectrometry/mass spectrometry (LC-MS/MS) analysis of excised gel bands led to the identification of seven subunits, from CSN-1 to CSN-7a, in the Myc-His-CSN-5 purified products and in the Myc-His-CSN-5H127A purified products (Figure 3C). Taken together, these results confirm that the integrity of the CSN complex is not affected by mutations within the JAMM motif of CSN-5 in *N. crassa*.

Next, we examined whether CSN complexes with mutant CSN-5 subunits can still interact with Cul1 protein. As shown in Figure 3D, both wild-type CSN-5 and each of the mutant CSN-5 proteins co-immunoprecipitated with Cul1 protein. We further examined whether CSN complexes with mutant CSN-5 subunits can also interact with Cul4 protein *in vivo* by IP/western blotting experiments. As shown in Figure 3E, the Myc-tagged wild-type CSN-5 co-immunoprecipitated with the neddylated and unneddylated Cul4, indicating that the *N. crassa* CSN complex can interact with all species of Cul4 *in vivo*. Similarly, the Myc-tagged mutant CSN-5s also co-precipitated with Cul4 (Figure 3E), further confirming that mutations within the JAMM metal-binding motif of CSN-5 do not interfere with interaction between CSN and cullins. These results strongly suggest that the point mutations within the JAMM metal-binding motif abolish NEDD8 isopeptidase activity but have no effect on the integrity of the CSN or on its interactions with cullins.

### CSN-5 with mutations in the metal-binding motif of JAMM domain can partially restore SCF-mediated FRQ degradation in the *csn-5^KO^* strain

**Figure 4:**
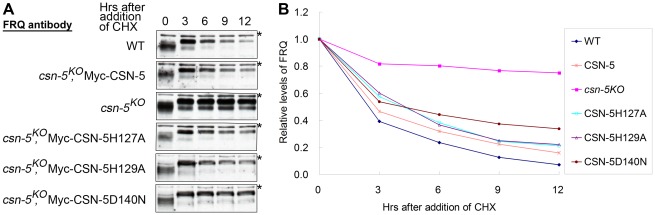
Mutations in the JAMM motif of CSN-5 partially restore SCF-mediated FRQ degradation in the *csn-5^KO^* strain. (A) Western blot analyses showing degradation of FRQ protein in *csn-5* mutant and the different CSN-5 complementation strains after addition of cycloheximide (10 mg/mL). Asterisks indicate nonspecific bands detected by FRQ antibody. (B) Densitometric analyses from four independent experiments showing the degradation of FRQ in different strains.

In *N. crassa*, the clock protein FREQUENCY (FRQ) is a negative regulator in the negative feedback loop that controls the circadian clock under constant conditions [34], [35]. Impaired FRQ degradation in *csn-2* mutants results in the loss of circadian rhythm [24]. To investigate whether the mutant CSN-5s can rescue circadian rhythm defects in the *csn-5^KO^* strain, we examined the degradation of FRQ protein in the wild-type and *csn-5^KO^* strains expressing wild-type CSN-5 or mutant CSN-5s after addition of the protein synthesis inhibitor cycloheximide (CHX). In the wild-type strain, the FRQ was gradually degraded after CHX treatment, with a half-life of about 2.5 h (Figure 4A and 4B). However, in the *csn-5^KO^* strain, the degradation of FRQ was mostly blocked (Figure 4A and 4B), similar to what was observed in the *csn-2^KO^* strain and the *fwd1^RIP^* mutant [24], [36]. As shown in Figure 4A and 4B, the expression of Myc-tagged wild-type CSN-5 in the *csn-5^KO^* strain restored the degradation of FRQ to wild-type levels, so that the conidiation period on race tubes was similar to that of the wild-type strain (Figure 2C). We next checked FRQ degradation in the *csn-5^KO^* strain expressing CSN-5 proteins with mutations in the JAMM metal-binding site. As shown in Figure 4A and 4B, the expression of Myc-tagged CSN-5H127A, CSN-5H129A, or CSN-5D140N in the *csn-5^KO^* strain partially rescued the degradation of FRQ in the *csn-5^KO^* strain. FRQ was degraded slightly slower in the mutants than the wild-type strain or the *csn-5^KO^* strain complemented by wild-type CSN-5, with a half-life of ∼5 h, consistent with the prolonged period of the conidiation rhythms in the *csn-5^KO^* strains expressing the mutant CSN-5, indicating that both deneddylation activity and integrity of CSN are needed in this process. Taken together, these results demonstrate that CSN-5 with point mutations in the JAMM metal-binding site partially restore the SCF-mediated FRQ degradation.

### Catalytically dead CSN-5 partially stabilizes the SCF^FWD-1^ complex

**Figure 5:**
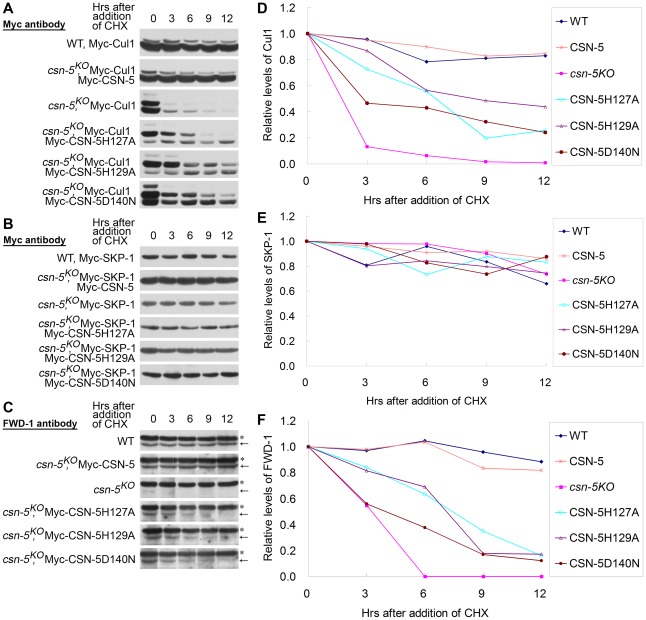
CSN-5–mutated CSN efficiently prevents degradation of components of the SCF^FWD-1^ complex. (A–C) Western blot analyses with labeled antibodies showing degradation of Myc-Cul1 (A), Myc-SKP-1 (B), and FWD-1 (C) after addition of cycloheximide (10 mg/mL) in the wild-type, *csn-5^KO^*, and different CSN-5 complementation strains. Arrows point out FWD-1 protein bands. Asterisks indicate nonspecific bands detected by FWD-1 antibody. (D–F) Densitometric analyses from four independent experiments showing the degradation of Myc-Cul1 (D), Myc-SKP-1 (E), and FWD-1 (F).

Previous studies showed that FRQ ubiquitination and degradation is mediated by the SCF^FWD-1^ E3 ligase complex [24], [36], and that the stability of E3 ligase components is controlled by CSN *in vivo* [3], [7], [16], [24]. Because the ectopic expression of mutated CSN-5 partially rescued both the circadian conidiation rhythm and FRQ degradation in the *csn-5^KO^* strain, we decided to check whether CSN with mutant CSN-5 can prevent the degradation of components of the SCF^FWD-1^complex. As shown in Figure 5A, Myc-Cul1 was stable after induced expression of Myc-CSN-5 in the *csn-5^KO^* strain, with a half-life of >9 h in the presence of CHX, similar to that of the wild-type strain. In the *csn-5* mutant, however, both the neddylated and unneddylated Myc-Cul1 became very unstable, with a half-life about 1.5 h (Figure 5A and 5D) [16]. Interestingly, the expression of JAMM mutant CSN-5 had a differential effect on the neddylated and unneddylated Cul1. In mutant CSN-5 transformants, the stability of neddylated Cul1 was only partially rescued, with a half-life of >3 h in the presence of CHX (Figure 5A and 5D), whereas the stability of unneddylated Cul1 was almost rescued, with a half-life of >12 h (Figure 5A and 5D). These data indicate that although CSN containing JAMM mutated CSN-5 fails to cleave NEDD8 from neddylated Cul1, it still functions to protect hyperneddylated and unneddylated Cul1 from degradation to a certain extent.

In *N. crassa*, deletion of *csn-5* or *csn-3* has no effect on the stability of SKP-1 protein in the SCF^FWD-1^ complex [16]. As expected, Myc-SKP-1 were very stable in the wild-type strain and the *csn-5^KO^* strain and in the complementation strains with mutant CSN-5, with a half-life of >12 h (Figure 5B and 5E).

FWD-1, the substrate-recruiting subunit of the SCF^FWD-1^ complex, was quite stable in the wild-type strain, whereas it became undetectable after only 3 h of CHX treatment in *csn-5^KO^* strain (Figure 5C and 5F). In the *csn-5^H127A^*, *csn-5^H129A^*, and *csn-5^D140N^* strains, however, FWD-1 signals could still be detected after 6 h of CHX treatment (Figure 5C and 5F), indicating that CSN with mutated CSN-5 partially functions to protect F-box proteins from degradation. This finding further confirms that regulation of SCF-mediated FRQ degradation by the CSN is a key step in the *N. crassa* circadian clock. Therefore, both the deneddylation activity and the integrity of the CSN are important for preventing the degradation of components of the SCF^FWD-1^ complex.

### CSN complex containing mutant CSN-5 efficiently prevents degradation of substrate receptors of CRLs

**Figure 6:**
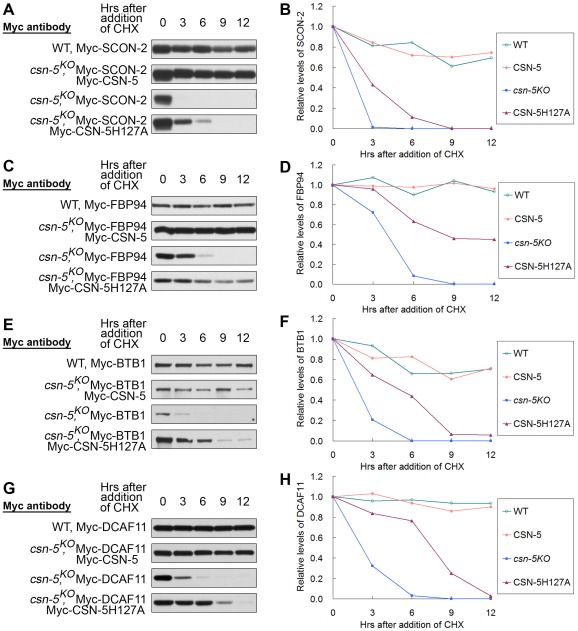
CSN containing mutant CSN-5 efficiently prevents degradation of substrate receptors of CRLs. Western blot analyses with labeled antibody showing degradation of Myc-SCON-2 (A), Myc-FBP94 (C), Myc-BTB1 (E), and Myc-DCAF11 (G) after addition of cycloheximide (10 mg/mL) in the wild-type, *csn-5^KO^*, *csn-5^KO^* complementation with CSN-5 and CSN-5H127A strains. Densitometric analyses from four independent experiments showing the degradation of Myc-SCON-2 (B), Myc-FBP94 (D), Myc-BTB1 (F), and Myc-DCAF11 (H).

We next asked whether CSN with mutated CSN-5 still functions to protect other CRL substrate receptors from degradation. *N. crassa* SCON-2, an F-box protein involved in regulating sulfur metabolism, was previously shown to interact with SKP-1 and is very unstable in a *csn-2^KO^* strain [24], [37]. We compared the stability of Myc-SCON-2 in wild-type, *csn-5^KO^* and *csn-5^KO^* expressing wild-type CSN-5 or mutant CSN-5H127A strains. The half-life of Myc-SCON-2 was approximately 12 h in the wild-type and *csn-5^KO^* expressing wild-type CSN-5 strains in the presence of CHX. Myc-SCON-2 was very unstable in the *csn-5* mutant and became undetectable after 3 h of CHX treatment (Figure 6A and 6B). In the *csn-5^H127A^* strain, the detection of Myc-SCON-2 signal extended to 6 h after CHX treatment (Figure 6A and 6B). FBP94 (NCU04785), another F-box–containing protein in *N. crassa*, can also interact with SKP-1 (data not shown). As shown in Figure 6C and 6D, FBP94 was quite stable in the wild-type strain and *csn-5^KO^* strain complemented with Myc-CSN-5, whereas in the *csn-5^KO^* strain it became undetectable after only 6 h of CHX treatment. In the *csn-5^H127A^* strain, detection of FBP94 signal extended to 12 h after CHX treatment (Figure 6C and 6D). Therefore, CSN complex with mutated JAMM domain can partially function in maintaining the stability of other F-box–containing adaptor proteins.

In a previous study, we determined that the *N. crassa* Cul3 protein interacts with BTB1 protein, and both proteins become unstable in the *csn-5^KO^* strain [16]. The half-life of Myc-BTB1 was >12 h in the wild-type and *csn-5^KO^* expressing wild-type CSN-5 strains in the presence of CHX, whereas in the *csn-5^KO^* strain it became undetectable after 6 h of CHX treatment (Figure 6E and 6F). As expected, in the *csn-5^H127A^* strain, BTB1 signals were detectable at 12 h after CHX treatment (Figure 6E and 6F), indicating that CSN with the JAMM mutated CSN-5 still partially functions to protect the substrate adaptor proteins of CRL3 from degradation. We also investigated whether CSN with the JAMM mutated CSN-5 regulates the substrate receptor protein of CRL4 in a similar manner. *N. crassa* Cul4 was previously shown to interact with DCAF11, a putative substrate receptor of CRL4^DCAF11^ [16], [38]. As shown in Figure 6G and 6H, the half-life of Myc-DCAF11 was >12 h in the wild-type and *csn-5^KO^* expressing wild-type CSN-5 strains in the presence of CHX, whereas in the *csn-5^KO^* strain it became undetectable after 6 h of CHX treatment [16]. As expected, in the *csn-5^H127A^* strain, the detection of DCAF11 signal was extended to 9 h after CHX treatment (Figure 6G and 6H). Taken together, these *in vivo* results indicate that the CSN complex containing mutant CSN-5 efficiently prevents degradation of substrate receptors of CRLs.

### CAND1 is not required for regulation of the circadian rhythm and SCF^FWD-1^ ubiquitin ligase

**Figure 7:**
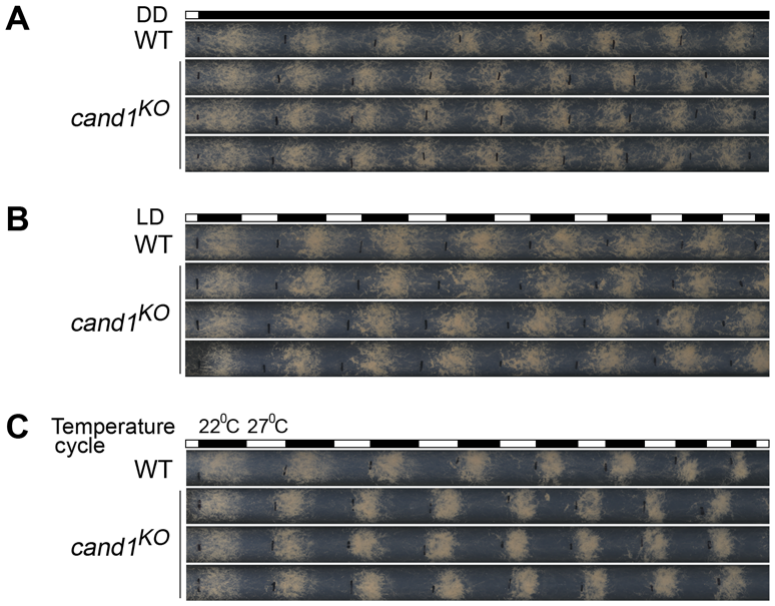
CAND1 is not required for regulation of the circadian rhythm. (A–C) Normal conidiation rhythms of *cand1* mutants in dark–dark (A), light–dark (B), and temperature cycles (C) measured by race tube assays. At least four replicates were tested under each condition. Black lines indicate the growth fronts every 24 h.

Current models suggest that the activity and assembly of CRLs are controlled by cycles of CRL deneddylation and CAND1 binding of deneddylated cullins [39], [40], [41], [42], [43], [44], [45], [46]. In plants and worms, CAND1 mutants exhibit defects consistent with a positive role in regulating the function of a subset of CRLs [40], [47], [48], [49]. However, in yeast and human cells, loss of CAND1 has little effect on the abundance of neddylated cullins, suggesting that the neddylation/deneddylation cycle may function independently of CAND1 [50], [51]. To test whether CAND1 is involved in maintaining the function of CRLs in *N. crassa*, we examined the role of CAND1 in the regulation of circadian conidiation rhythm and proper functioning of the SCF^FWD-1^ complex. We first measured the growth rates of the wild-type and *cand1^KO^* strains by race tube assay under constant darkness. The growth of the *cand1^KO^* strain (about 3.0 cm per day) was slightly slower than that of the wild-type strain (about 3.7 cm per day), suggesting that CAND1 is involved in regulating hyphal growth. After entrainment by light, like the wild-type strain, the *cand1^KO^* strain exhibited a robust circadian conidiation rhythm with a period of about 22 h at 25°C in constant darkness (Figure 7A), suggesting that CAND1 is not required for circadian rhythms in *N. crassa*. To test whether CAND1 functions in a manner similar to the CSN, we examined the conidiation rhythms of the *cand1* mutant in LD cycles (12 h light/12 h dark). As shown in Figure 7B, the conidiation rhythms of the *cand1^KO^* strain were entrained by LD cycles, indicating that unlike CSN, CAND1 is not required in light regulation of the circadian clock. We also examined the responses of the *cand1* mutant to temperature entrainment using race tube assays. As expected, in 12 h 27°C/12 h 22°C temperature cycles, as shown in Figure 7C, like the wild-type strain, the conidiation rhythm of the *cand1^KO^* strain was synchronized by the temperature cycles, indicating that CAND1 is not required for the temperature-entrained conidiation process. These results suggest that CAND1 does not play a significant role in the regulation of circadian rhythm in *N. crassa*.

**Figure 8:**
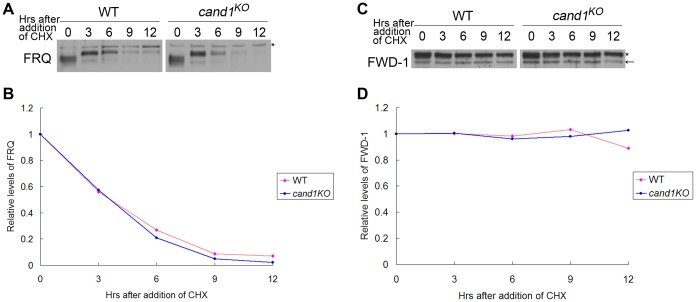
CAND1 is not required for regulation of the SCF^FWD-1^ ubiquitin ligase. Western blot analyses showing degradation of FRQ (A) and FWD-1 (C) in wild-type and *cand1^KO^* strains after addition of cycloheximide (10 mg/mL). Densitometric analyses from four independent experiments showing the degradation of FRQ (B) and FWD-1 (D). Arrows point out FWD-1 protein bands. Asterisks indicate nonspecific bands detected by FRQ antibody (A) or FWD-1 antibody (C).

Deletion of *cand1* also had no effect on degradation of the clock protein FRQ, which is the substrate of the SCF^FWD-1^ ubiquitin ligase complex in *N. crassa* (Figure 8A and 8B). We also examined the stability of FWD-1 of the SCF^FWD-1^ complex in the *cand1^KO^* strain. As shown in Figure 8C and 8D, FWD-1 was very stable in the *cand1* mutant, as in the wild-type strain, with a half-life of >12 h. Together, these results suggest that CAND1 is not required for regulation of the circadian rhythm and for maintaining the proper function of the SCF^FWD-1^ complex in *N. crassa*.

## Discussion

In eukaryotes, the COP9 signalosome (CSN) is a highly conserved multifunctional complex that has two major biochemical roles: cleaving NEDD8 from cullin proteins [1], [3], [7], [11] and maintaining the stability of the CRL components [7], [24]. In this study, we used mutation analysis to confirm that the JAMM metal-binding motif of the CSN-5 subunit is responsible for NEDD8 cleavage from cullin proteins in *N. crassa*. Point mutations of the key residues in the metal-binding motif (EX_n_ HXHX_10_ D) of the CSN-5 disrupted CSN deneddylation activity without interfering with the CSN assembly. We demonstrated that those mutant CSN-5s could almost restore the growth and conidiation defects of the *csn-5^KO^* strain. Furthermore, even without the deneddylation activity, the CSN partially maintained the stability of the SCF^FWD-1^ complex and partially restored the degradation of clock protein FRQ *in vivo*. Finally, we also showed that CSN containing mutant CSN-5 could efficiently prevent the degradation of the substrate receptors of CRLs. In addition, deletion of the CAND1 ortholog in *N. crassa* had little effect on the circadian rhythm of conidiation. Thus, our results suggest that maintenance of CRL stability by the CSN integrity is even more crucial in hyphal growth, conidial development, and circadian function in *N. crassa*.

### Both deneddylation activity and integrity of CSN are required for maintenance of CRL stability

As the key regulator of CRLs, both deneddylation and maintenance of CRL stability by the CSN occurs when the CSN binds to CRLs. Thus, it is difficult to distinguish which function is more important for maintaining the proper function of CRLs in eukaryotes. To precisely determine the function of the CSN in maintaining the stability of CRLs, we sought to separate the two functional aspects of CSN from one other in *N. crassa*. In those *csn-5^KO^* strains expressing CSN-5 proteins with different point mutations in the JAMM metal-binding motif, the deneddylation activity was disrupted, while the assembly of the CSN complex and interactions between CSN and cullin proteins were not affected. Therefore, this system has great potential as a model for distinguishing between the two activities of the CSN.

A recent study suggests that neddylated Cul1 and Cul3 are unstable in *D. melanogaster csn* mutant cells due to a defect in CSN deneddylation activity, whereas unneddylated cullins are stable in *csn-5* mutant cells [33]. The results presented here show that unstable forms of Cul1 in the *csn-5* mutant were partially restored by expression of mutant CSN-5 protein without deneddylation activity. Like the stability of unneddylated Cul1 and Cul3 in *D. melanogaster* CSN-5–defective cells [33], unneddylated Cul1 remained stable in the *csn-5^H127A^*, *csn-5^H129A^*, and *csn-5^D140N^* strains, similar to that in the wild-type strain (Figure 5A), indicating that CSN integrity with catalytically dead CSN-5 effectively maintains the stability of cullins. Studies in *D. melanogaster* and *A. nidulans* CSN-5 mutants indicated that the CSN deneddylation activity is essential for cell differentiation and developmental initiation [11], [19], [22], [33]. However, in the *A. thaliana fus6*/*C231* mutant (a CSN1 N-terminal deletion mutant), although the Cul1 neddylation still works in a wild-type pattern, it was lethal and exhibited severe gene expression defects [52]. This genetic evidence raises questions concerning whether the CSN has other important functions aside from its deneddylation activity. The accelerated degradation of c-Jun in HeLa cells in which CSN-5 is downregulated is rescued equally by over-expression of the deneddylation mutant CSN-5D151N or wild-type CSN-5 [20]. These data suggest that two activities of CSN may function parallel for regulating the activity of CRLs.

Bennett *et al.* found that Cul1K720R (a constitutively unneddylated Cul1 mutant) assembles with CSN, SKP-1, and most F-box proteins to the same extent as wild-type Cul1 [51]. Our IP experiments also show that wild-type CSN interacts with both neddylated and unneddylated Cul4. These findings suggest that the CSN can interact with CRLs independent of the prior neddylation of cullins. In plants, genetic results also suggest that during early embryo development and germination, neddylation/deneddylation cycling is not absolutely required, although it becomes more important during seedling establishment and later in development [21], suggesting that the CSN has distinct biochemical functions that orchestrate development in the appropriate spatial and temporal setting.

Protection of substrate receptors by the CSN has been described for the CRLs *in vivo* [24], [25], [26], [27], [28]. We found that CSN with mutated CSN-5 had a contribution to the stabilities of five receptor proteins of CRLs *in vivo*. These results provide evidence for the idea that the abundance of adaptor modules (rather than cycles of neddylation/deneddylation and CAND1 binding) drives CRL network organization [51]. This possibility is supported by our genetic observations that the *csn-5^H127A^*, *csn-5^H129A^*, and *csn-5^D140N^* strains exhibited normal growth and conidiation phenotypes. The integrity of CSN was maintained in these JAMM mutation complementation strains, thus it can serve as a platform to recruit other proteins for regulating the activities of CRLs, such as the recruitment of UBP12 in yeast, as well as USP15 in human [14], [53]. In addition, a non-catalytic CSN itself may stabilize the substrate receptors of CRLs. A very recent study has shown that the protective effect of the CSN on DDB2 and CSA autoubiquitination is independent of CSN-5 mediated deneddylation *in vitro* [29]. These results suggest that the partial rescue of stability of substrate receptors by the catalytically dead CSN is mainly dependent on its protective effect. Therefore, the stability of cullins and some substrate receptors of CRLs are dependent on both deneddylation activity and integrity of the CSN in *N. crassa*.

### Maintaining stability of the SCF^FWD-1^ complex is the key process in circadian rhythm regulation

The *csn-5^KO^* strain exhibits abnormal conidiation rhythms in DD, which cannot be entrained by either LD or temperature cycles, indicating that light and temperature regulation of the conidiation process is impaired in this mutant [16]. We found that degradation of the clock protein FRQ is impaired in the *csn-5^KO^* strain, especially when protein synthesis is completely blocked. To further characterize the molecular mechanism of how the CSN regulates the conidiation rhythm, we focused on the SCF^FWD-1^ ubiquitin ligase, which controls the *N. crassa* circadian rhythm by ubiquitinating FRQ [36]. Our results demonstrated that defective FRQ degradation in the *csn-5^KO^* strain is due to the drastically reduced stability and levels of FWD-1 and Cul1 proteins in the SCF^FWD-1^ complex. Ectopic expression of mutant CSN-5 without deneddylation activity restored the defects of growth and conidiation in the *csn-5^KO^* strain, and almost restored the defects of the circadian conidiation rhythm in DD and FRQ degradation in the *csn-5^KO^* strain. Our data further showed that the low levels of FWD-1 in the *csn-5^KO^* strain were dramatically increased after expression of each of the CSN-5 proteins with point mutations in the JAMM metal-binding site, however, the increased stability and levels of the components in the SCF^FWD-1^ ubiquitin ligase are not enough to fully restore the degradation of FRQ to wild-type level, indicating that regulation of FRQ degradation plays a key role in maintaining the precise period length of conidiation rhythm in *N. crassa*. This is further supported by the finding that accelerated degradation of c-Jun in HeLa cells in which CSN-5 is downregulated can be rescued equally by over-expression of the deneddylation mutant CSN-5D151N or wild-type CSN-5; however, accelerated c-Jun degradation is not rescued in CSN-1– or CSN-3–downregulated cells by over-expression of wild-type CSN-5 [20]. Furthermore, the degradation of EB1 (microtubule-end-binding protein 1) is accelerated by over-expression of wild-type CSN-5 or CSN-5D151N in HeLa cells [20]. These results suggest that the integrity of CSN might contribute more to regulating the stability of some substrates of CRLs. Current models suggest that the CRL complex is controlled by cycles of CRL deneddylation and CAND1 binding [7]. Our experiments further suggested that CAND1, a putative regulator of CRLs, is not required for maintenance of SCF^FWD-1^ ubiquitin ligase activity and circadian rhythm in *N. crassa*. These data provide additional evidence that the CSN is an important regulator of the circadian clock in *N. crassa* through maintenance of SCF^FWD-1^ ubiquitin ligase stability.

In conclusion, the results of our experiments indicate that even without deneddylation activity, the *N. crassa* CSN can still regulate hyphal growth, conidial development, and circadian function by regulating the activities of E3 ubiquitin ligases. Because the function of the CSN in the regulation of CRL activities is conserved in higher eukaryotes, we propose that the CSN may have a similar role in plants and animals.

## Materials and Methods

### Strains and culture conditions

The *N. crassa* strain 87-3 (*bd*, *a*) was used as the wild-type strain in this study. The *bd ku70^RIP^* strain, which was generated previously [54], was used as the host strain for creating the *cand1* knockout mutants. We also used *csn-5^KO^*, *csn-2^KO^* and *csn-5^KO^*, *his-3* strains that were generated previously [16]. The 301-6 (*bd, his-3, A*) strain and the *csn-5^KO^, his-3* strain were used as the host strains for the *his-3* targeting construct transformation [24]. Liquid culture conditions were the same as described previously [34]. For QA-induced protein expression, 0.01 M QA (pH 5.8) was added to liquid medium containing 1× Vogel's medium, 0.1% glucose, and 0.17% arginine. The medium for the race tube assay contained 1× Vogel's, 0.1% glucose, 0.17% arginine, 50 ng/mL biotin, and 1.5% agar [55]. For race tubes containing QA (10^−3^ M), glucose was excluded from the medium.

### Plasmids

All three JAMM point mutations of CSN-5 were generated using the Quikchange Site-Directed Mutagenesis Kit (Stratagene). pUC19-CSN-5 was used as the template for mutagenesis. Afterwards, the mutated CSN-5 DNA fragments were subcloned into either the pqa-5Myc-6His or pqa-3Flag vectors. The triple point mutant CSN-5 (H127A, H129A and D140N) generated from pUC19-CSN-5 was subcloned into the endogenous *csn-5* promoter-driven vector pcsn-5-Myc-His-CSN-5, resulting in pcsn-5-Myc-His-CSN-5tri. The previously constructed plasmids pqa-Myc-Cul1, pqa-Myc-His-Cul3, pqa-Myc-His-Cul4, pqa-Myc-His-CSN-6, pqa-Myc-His-SCON-2, pqa-Myc-His-FBP94, pqa-Myc-His-BTB1, and pqa-Myc-His-DCAF11 were also used for *his-3* targeting transformation in the *csn-5^KO^, his-3* and 301-6 (*bd, his-3, A*) strains [16] and cotransformation in the *csn-5^H127A^*, *csn-5^H129A^*, and *csn-5^D140N^* strains.

### Generation of antiserum against Cul4

GST-Cul4 (containing Cul4 amino acids 1–113) fusion protein was expressed in RIL cells and the recombinant protein was purified and used as the antigen to generate rabbit polyclonal antiserum, as described previously [56].

### Purification of Myc-His-CSN-5 and mutant Myc-His-CSN-5 proteins from *N. crassa*

The *csn-5^KO^*, Myc-His-CSN-5H127A, *csn-5^KO^*, Myc-His-CSN-5H129A, or *csn-5^KO^*, Myc-His-CSN-5D140N strain, wild-type strain (negative control), and *csn-5^KO^*, Myc-His-CSN-5 strain (positive control) were cultured for approximately 24 h in constant light (LL) in liquid medium containing QA (0.01 M QA, 1× Vogel's medium, 0.1% glucose, and 0.17% arginine). Approximately 10 g of tissue from each strain grown in LL was harvested. The purification procedure was the same as described previously [16]. Fractions containing purified Myc-tagged CSN proteins were immunoprecipitated by adding 25 µL of c-Myc monoclonal antibody-coupled agarose beads (9E10AC, Santa Cruz Biotechnology). The precipitates of each sample were analyzed by SDS-PAGE (4%–20% acrylamide), which was subsequently silver stained following the manufacturer's instructions (ProteoSilver Plus, Sigma). Specific bands in the Myc-His-CSN-5 purified products or in the Myc-His-CSN-5H127A purified products were excised and subjected to tryptic digestion and LC-MS/MS.

### Gel filtration chromatography of Myc-His-CSN-5 or mutant CSN-5s in *csn-5* mutant

The protocol of gel filtration chromatography was the same as described previously [16], [21]. Briefly, purified proteins (400 µg) were loaded onto a Superdex™ 200 (GE) gel filtration column that was equilibrated with 25 mL (150 mM NaCl, 20 mM Tris Cl pH 7.4). The proteins were eluted in the same buffer at a flow rate of 0.3 mL/min. Fractions of 0.4 mL were collected starting from the onset of the column void volume (8.0 mL) and finishing at 18 mL (25 fractions). 20 µL of each fraction were prepared in 20 µL of 2× SDS loading buffer, separated by 7.5% SDS-PAGE, and then examined by Western blot analysis using c-Myc antibody (9E10, Santa Cruz Biotechnology).

### Protein analyses

Protein extraction, quantification, western blot analysis, protein degradation assays, and immunoprecipitation assays were performed as described previously [24], [56]. Western blot analyses using a monoclonal c-Myc antibody (9E10, Santa Cruz Biotechnology) or Flag antibody (F3165-5MG, Sigma) were performed to identify the positive transformants. Immunoprecipitates or equal amounts of total protein (40 µg) were loaded into each protein lane for SDS-PAGE. After electrophoresis, proteins were transferred onto a PVDF membrane, and western blot analysis was performed using c-Myc antibody, Flag antibody, FWD-1 antiserum, FRQ antiserum, or Cul4 antiserum.

## Supporting Information

Figure S1 Expression of Myc-Cul1 in the first generation of *csn-1^KO^*, *csn-5^KO^*, and *csn-6^KO^* transformants. The positive transformants showing the expression profile of Myc-Cul1 in the *csn-1^KO^*, *csn-5^KO^*, and *csn-6^KO^* strains. Western blot analysis was performed using c-Myc antibody. Note that the total protein loaded into each lane was not quantified for identifying positive transformants.
(TIF)
