# Correction: *Neurospora* COP9 Signalosome Integrity Plays Major Roles for Hyphal Growth, Conidial Development, and Circadian Function

**DOI:** 10.1371/annotation/b22478ae-9e5d-46fc-8b88-f1cd0cb9a158

**Published:** 2012-05-18

**Authors:** Zhipeng Zhou, Ying Wang, Gaihong Cai, Qun He

In the Funding section, the grant number from the funder National Basic Research Program of China (973 Program) was incorrect. The correct grant number is: 2012CB947600.

