# Supplementary figures and images for: Neurospora COP9 Signalosome Integrity Plays Major Roles for Hyphal Growth, Conidial Development, and Circadian Function
Source: PLoS Genet. 2012 May 10;8(5):e1002712. doi: 10.1371/journal.pgen.1002712 (PMC3349749; doi:10.1371/journal.pgen.1002712)

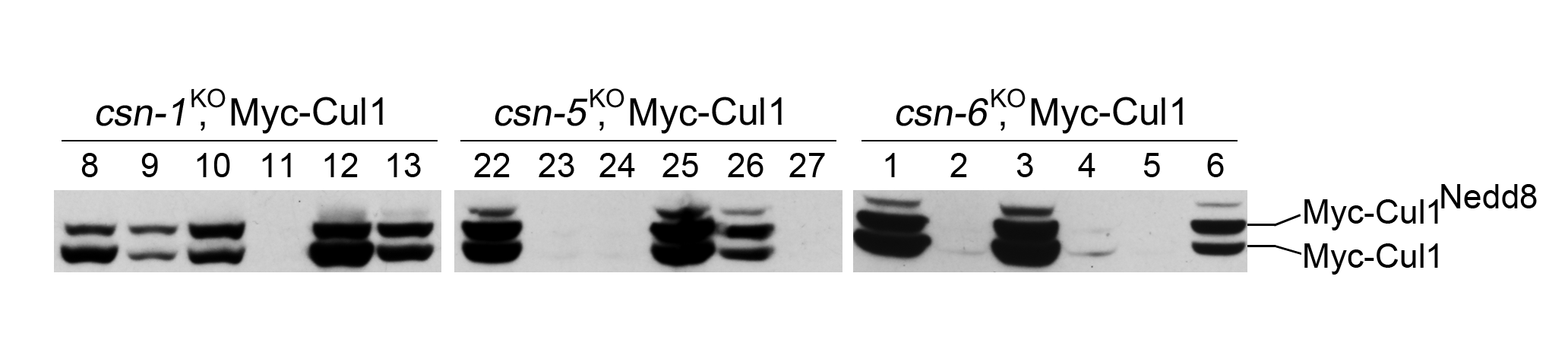

Supplement: Figure S1 — Expression of Myc-Cul1 in the first generation of csn-1KO, csn-5KO, and csn-6KO transformants. The positive transformants showing the expression profile of Myc-Cul1 in the csn-1KO, csn-5KO, and csn-6KO strains. Western blot analysis was performed using c-Myc antibody. Note that the total protein loaded into each lane was not quantified for identifying positive transformants. (TIF) [file pgen.1002712.s001.tif]
